# Postprandial Glucose as a Risk Factor for Elevated Intraocular Pressure

**DOI:** 10.1371/journal.pone.0168142

**Published:** 2016-12-15

**Authors:** Chen-Jung Wu, Wen-Hui Fang, Tung-Wei Kao, Ying-Jen Chen, Fang-Yih Liaw, Yaw-Wen Chang, Gia-Chi Wang, Tao-Chun Peng, Wei-Liang Chen

**Affiliations:** 1 Division of Family Medicine, Department of Family and Community Medicine, Tri-Service General Hospital; and School of Medicine, National Defense Medical Center, Taipei, Taiwan; 2 Division of Family Medicine, Department of Community Medicine, Taoyuan Armed Forces General Hospital, Taoyuan, Taiwan; 3 Division of Geriatric Medicine, Department of Family and Community Medicine, Tri-Service General Hospital; and School of Medicine, National Defense Medical Center, Taipei, Taiwan; 4 Graduate Institute of Clinical Medical, College of Medicine, National Taiwan University, Taipei, Taiwan; 5 Department of Ophthalmology, Tri-Service General Hospital; and School of Medicine, National Defense Medical Center, Taipei, Taiwan; 6 Graduate Institute of Medical Sciences, National Defense Medical Center, Taipei, Taiwan; Medical University Innsbruck, AUSTRIA

## Abstract

The aim of this study was to investigate the association between postprandial glucose and intraocular pressure in a relatively healthy population. We examined 1,439 adults getting a health check-up in a health promotion center at Tri-Service General Hospital (TSGH) in Taiwan between 2012 and 2013. All participants underwent examinations to measure metabolic variables and intraocular pressure. Multiple linear regression analyses were performed to assess the relationship between postprandial glucose and intraocular pressure. The levels of postprandial glucose were divided into quartiles with subjects in the lowest quartile being regarded as the reference group to perform quartile-based analysis. Covariate adjustment was designed for three models for further analysis. Subjects with higher quartiles of postprandial glucose level had a higher systolic blood pressure, a greater waist circumference and an elevated fasting glucose level (all p < 0.001). The β coefficient with adjusted covariates showed a significant positive association between postprandial glucose and intraocular pressure. The trends of intraocular pressure across increasing quartiles of postprandial glucose were statistically significant (all p for trend < 0.001). Thus, higher levels of postprandial glucose positively correlated with elevated intraocular pressure.

## Introduction

Elevated intraocular pressure (IOP) is identified as an amendable main compounding factor for glaucoma [1–3], which was regarded as the leading causes of permanent blindness worldwide according to a recent World Health Organization survey [4]. Therefore, the most important aspect of the management of glaucoma is the proper determination of individual IOP values.

Emerging evidence has illustrated the positive association of elevated IOP with a number of predisposing factors or chronic diseases, including age [5], sex [6], blood pressure [7,8], genetic linkage [9], central corneal thickness [10], arterial stiffness [11], diabetes [12] and obesity [13]. Among these predisposing factors that affect the intraocular pressure, we focused more on the metabolic variables due to their association with many systemic diseases including cardiovascular disease and diabetes [14,15], which are curable via life style modification and medication. In a cross sectional study involving 13,431 adults aged greater than 19 years in Korea, IOP was correlated significantly with the components of metabolic syndrome including body mass index (BMI), systolic blood pressure and fasting plasma glucose [16]. Furthermore, a cross sectional study involving 14,003 healthy Japanese participants aged 18 to 83 years also indicated that metabolic syndrome was a risk factor for elevated IOP [17]. According to a recently published meta-analysis in the *American Academy of Ophthalmology*, impaired fasting glucose was a principal risk factor for elevated IOP among the components of metabolic syndrome [12]. However, previous studies have not put much more emphasis on postprandial glucose, which we regard as an unique measure of glycemic control, and which can be applied in the diagnosis of diabetes. Previous literature has indicated that postprandial glucose level is a better predictor of cardiovascular risk than HbA1c and fasting glucose levels [18]. Much remains to be understood regarding the pathophysiologic effect of postprandial glucose in other disorders. The association between postprandial glucose and IOP has not been investigated extensively; therefore, the aim of this study was to clarify this potential relationship.

A review of the literature indicated that data on the level of fasting plasma glucose used as one of the covariates in association analysis for IOP in patients with metabolic syndrome and diabetes were inconsistent. This observation prompted us to clarify the possible correlation between metabolic abnormalities of postprandial glucose and elevated IOP. Therefore, the purpose of the present study was to investigate the effects of postprandial glucose on IOP in a healthy population.

## Materials and Methods

### Study Subjects

**Fig 1:**
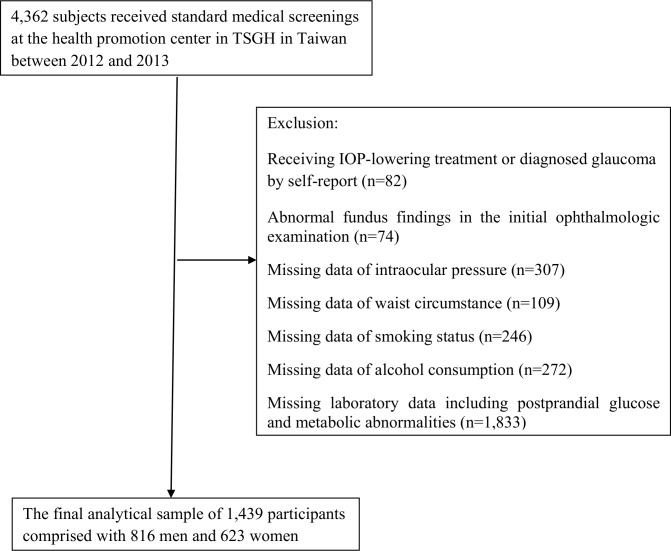
Flowchart for subjects selection.

Data were obtained from standard medical screenings at the health promotion center in Tri-Service General Hospital (TSGH) in Taiwan between 2012 and 2013. It was approved by the Institutional Review Board of TSGH in accordance with the revised Helsinki Declaration. This is a retrospective analysis of the patients who underwent health check-up exams, without disclosure of their identity. The Institutional Review Board waived the need to obtain individual informed consent because the data were analyzed anonymously. Some subjects completed health check-up more than once during this period, but only the initial visit was used in our study. The demographic characteristics and lifestyle information were collected from a self-assessment questionnaire. We excluded participants who received IOP-lowering treatment or diagnosed glaucoma by self-report (n = 82); abnormal fundus findings in the initial ophthalmologic examination (n = 74); missing survey data regarding IOP (n = 307), waist circumstance (n = 109), smoking status (n = 246), alcohol consumption (n = 272) and laboratory data including postprandial glucose and metabolic abnormalities (n = 1,833). The final analytical sample of 1,439 participants comprised with 816 men and 623 women (Fig 1).

### Laboratory Examinations

The standard physical examination program including blood testing was commenced after 8 to 10 hours of overnight fast to obtain serum levels of total cholesterol, high-density lipoprotein cholesterol, triglyceride and fasting glucose. To obtain postprandial glucose values, all participants consumed a standard 600-kcal mixed meal (57% carbohydrate, 24% fat and 19% protein) within 30 minutes of 11 A.M. after completing the blood sample collection and laboratory analyses. Postprandial glucose was measured 2 hours after commencement of the meal (1 P.M.). The measurement of capillary glucose was performed by collecting blood from the patient’s finger by piercing the skin using a needle and syringe. A previous study indicated a close correlation between different glucometers and laboratory values including one-touch examination [19].

### Ophthalmological Examination

The examination protocols at our health promotion center in TSGH include ophthalmologic examinations conducted by ophthalmologists. All subjects with abnormal fundus findings such as retinopathy or an elevated C/D ratio in the initial examinations received further testing to exclude the possibility of glaucoma. These subjects were excluded at the beginning of the study. Thus, we reduced the likelihood of enrolling glaucoma patients in our study as much as possible. Intraocular pressure was measured at the center of the cornea once for each eye from left to right by TOPCON CT-80 non-contact tonometry (Abdulrehman Al-Gosaibi GTB, Riyadh, Saudi Arabia). To avoid the effects of diurnal variation, we performed the measurement between 8 and 10 A.M. for all participants. All measurements were taken with the participants in a sitting position by trained ophthalmologists. We excluded subjects who were receiving an IOP-lowering treatment or who had a self-reported diagnosis of glaucoma. After measurement of the IOP values, mean IOP values for bilateral eyes were calculated for the regression analysis.

### Covariates

Age, sex, smoking history and alcohol consumption were obtained by self-report and identity card. Body weight and height were obtained using a digital scale, and BMI was calculated according to the formula that divided the subject’s weight in kilograms by the square of the height in meters (kg/m^2^). Waist circumference was measured in the standing position using a flexible, non-elastic anthropometric tape to the nearest 0.1 centimeter. The standard protocol to measure blood pressure in our study was placing the cuff on the right upper arm with the subjects in a sitting position after 10 to 15 minutes rest using a digital automatic sphygmomanometer after blood sampling with the exception of using the right arm in some special status. The definition of diabetes was based on either a self-reported medical diagnosis or a fasting plasma glucose level greater than or equal to 126  mg/dL obtained during the health check-up examination.

### Statistical Analysis

All data analysis was performed by Predictive Analytics Suite Workstation Statistics version 18.0 (SPSS Inc, Chicago, IL). The components of metabolic syndrome (fasting glucose, systolic blood pressure, serum cholesterol, etc) were regarded as continuous variables. The other covariates were defined as categorical variables including gender and smoking history. The level of postprandial glucose was divided into quartiles and the participants belonging to the lowest quartile were regarded as the baseline group with a view to performing quartile-based analysis. The cutoff postprandial glucose levels for the quartiles were as follows: Q1 < 112.0 mg/dL, 112.0 ≤ Q2 < 127.0 mg/dL, 127.0 ≤ Q3 < 152.0 mg/dL, and Q4 ≥ 152.0 mg/dL. We investigated the correlation between postprandial glucose and intraocular pressure using linear regression analysis. Covariate adjustments were designed for the following three models: Model 1  =   gender, age; Model 2  =   Model 1+  history of diabetes mellitus, alcohol consumption, and smoking; Model 3  = Model 2+ BMI and the factors of metabolic syndrome (waist circumference, fasting glucose, systolic blood pressure, total cholesterol, high-density lipoprotein cholesterol and triglyceride).

## Results

### Characteristics of the Study Population

The study group consisted of 1,439 participants; 56.7% were men and the mean subject age was 51.53 ± 12.35 years old. Table 1 shows the characteristics of all subjects classified by quartiles of postprandial glucose level. Higher systolic blood pressure, higher waist circumference and fasting glucose level were prominent in the higher quartiles of postprandial glucose level compared with the lowest quartile of postprandial glucose level.

**Table 1:** Characteristics of the Study Participants.

	Quartiles of Postprandial Glucose Level					
Variables	Q1(<112.0)	Q2(112.0 to<127.0)	Q3(127.0 to<152.0)	Q4(>152.0)	Total	*P*
	(N = 368)	(N = 354)	(N = 364)	(N = 353)	(N = 1439)	value
Continuous variables ^a^						
Age, years	46.00(11.99)	48.98(11.68)	53.16(11.88)	58.17(10.24)	51.53(12.35)	<0.001
SBP, mm-Hg	116.41(16.25)	118.74(18.02)	122.26(19.70)	126.94(19.99)	121.04(18.94)	<0.001
Body mass index, kg/m^2^	24.11(4.05)	24.16(3.40)	24.58(3.77)	24.86(4.36)	24.43(3.92)	0.030
Waist circumference, cm	80.79(11.79)	81.79(10.19)	83.15(10.78)	83.93(10.15)	82.40(10.82)	<0.001
Serum cholesterol, mg/dL	192.82(37.42)	197.82(35.34)	194.70(37.40)	194.78(37.32)	195.01(36.89)	0.335
Serum triglyceride, mg/dL	135.26(83.26)	138.03(80.46)	147.46(153.83)	158.19(114.25)	144.65(112.28)	0.027
Serum HDL-C, mg/dL	52.45(15.71)	53.01(16.16)	51.17(15.77)	51.63(15.70)	52.06(15.83)	0.408
Fasting glucose, mg/dL	89.92(8.59)	91.25(9.22)	94.49(12.28)	115.94(37.88)	97.79(23.22)	<0.001
Categorical variables ^b^						
Male	209(25.6)	197(24.1)	218(26.7)	192(23.5)	816(56.7)	0.487
Diabetes mellitus	2(1.6)	2(1.6)	11(8.9)	108(87.8)	123(8.5)	<0.001
Alcohol consumption	204(28.1)	187(25.8)	179(24.7)	156(21.5)	726(50.5)	0.017
Smoking history	143(27.1)	143(27.1)	129(24.5)	112(21.3)	527(36.6)	0.078

SBP, systolic blood pressure; Serum HDL-C, serum high density lipoprotein cholesterol.

^a^ Values were expressed as mean (standard deviation)

^b^ Values in the categorical variables were expressed as number (%)

### Association between Postprandial Glucose and Intraocular Pressure

The postprandial glucose values revealed significant association with the level of intraocular pressure. The results of linear regression analyses are shown in Table 2. The adjusted β coefficient of intraocular pressure was 0.010 (95% confidence interval, 0.007–0.013, p<0.001) after adjustments for age and gender. Additional adjustment for all covariates did not affect the statistical significance in our study (β coefficient = 0.007, p<0.001). Table 3 showed the outcome of multiple linear regression analyses of postprandial glucose quartiles, which showed positive associations between postprandial glucose and intraocular pressure. Participants in the higher quartiles of postprandial glucose level had significantly higher intraocular pressure (p for trend<0.001).

**Table 2:** Association between Postprandial Glucose Level and the Level of Intraocular Pressure.

Models ^a^	β ^b^ (95% CI)	*P* value
Model 1	0.010 (0.007–0.013)	<0.001
Model 2	0.009 (0.005–0.012)	<0.001
Model 3	0.007 (0.003–0.011)	<0.001

SBP, systolic blood pressure; WC, waist circumference; Serum HDL-C, serum high density lipoprotein cholesterol.

^a^ Adjusted covariates

Model 1 = age, sex.

Model 2 = Model 1+ (history of diabetes mellitus, smoking, alcohol consumption)

Model 3 = Model 2+ (SBP, BMI, WC, serum cholesterol, serum triglyceride, serum HDL-C, fasting glucose)

^b^ β coefficients was interpreted as change of postprandial glucose level for each increase in intraocular pressure

**Table 3:** Association between Postprandial Glucose Level and Intraocular Pressure.

Models ^a^	Postprandial Glucose Level Quartiles	β ^b^ (95% CI)	*P* value	*P* for trend
Model 1	• Q2 v.s. Q1 • Q3 v.s. Q1 • Q4 v.s. Q1	• 0.763 (0.353, 1.172) • 0.871 (0.458, 1.285) • 1.357 (0.922, 1.792)	• <0.001 • <0.001 • <0.001	<0.001
Model 2	• Q2 v.s. Q1 • Q3 v.s. Q1 • Q4 v.s. Q1	• 0.768 (0.360, 1.176) • 0.865 (0.452, 1.277) • 1.059 (0.595, 1.523)	• <0.001 • <0.001 • <0.001	<0.001
Model 3	• Q2 v.s. Q1 • Q3 v.s. Q1 • Q4 v.s. Q1	• 0.728 (0.329, 1.126) • 0.805 (0.401, 1.209) • 0.897 (0.439, 1.354)	• <0.001 • <0.001 • <0.001	<0.001

SBP, systolic blood pressure; WC, waist circumference; Serum HDL-C, = serum high density lipoprotein cholesterol.

^a^ Adjusted covariates

Model 1 = age, gender.

Model 2 = Model 1+ (history of diabetes mellitus, smoking, alcohol consumption)

Model 3 = Model 2+ (SBP, BMI, WC, serum cholesterol, serum triglyceride, serum HDL-C, fasting glucose)

^b^ β coefficients was interpreted as change of postprandial glucose level for each increase in intraocular pressure

## Discussion

In this study, we investigated the association between the intraocular pressure and postprandial glucose. The most prominent finding of our study was that individuals with a higher quartile of postprandial glucose had a higher intraocular pressure compared with those with a lower quartile of postprandial glucose level, even after adjusting for compounding factors, which were identified as having a significant association with intraocular pressure in other trials. To the best of our knowledge, this study is the first to assess the relationship between postprandial glucose level and intraocular pressure in a healthy population.

We utilized three models to adjust for different covariates associated with IOP as mentioned in the previous section. Model 1 was adjusted for demographic variables including age and gender, which have been reported as predisposing factors for IOP [5,6]. Model 2 was adjusted for history of diabetes mellitus and lifestyle variables. Model 3 was adjusted for BMI and the components of metabolic syndrome, which have been reported as risk factors for elevated IOP [16,17].

The diabetes status correlated with elevated IOP in earlier reports [12,16] and fasting glucose levels in diabetic patients were associated with elevated IOP that increased the risk for developing glaucoma in an earlier study. However, the mechanisms relating these factors to increased IOP are unclear [12,20]. The possible reasons for the connection of high fasting glucose with elevated IOP may be due to a hyperglycemia induced osmotic gradient causing excessive fluid to shift into the anterior chamber [21]. Other research has reported that hyperglycemia may impair the function of the trabecular meshwork, leading to increased IOP [22]. For diabetic patients in this study, the glycemic control was regarded as the simplest way to determine the efficiency of the pharmacotherapy of outpatients. It was assessed by measuring glycated hemoglobin, postprandial plasma glucose and fasting plasma glucose. HbA1c is the most widely used tool and is regarded the gold standard for outpatients with diabetes. In addition, a capillary blood test for fasting plasma glucose was also performed during regular follow-ups when patients returned to the hospital for continuous prescription. In other words, postprandial plasma glucose was less used in the clinical assessment of glycemic control. A systematic review study compared fasting glucose and postprandial glucose to determine which provided a better prediction of glycemic control if HbA1c was not obtainable. The result showed that postprandial glucose had a closer relationship with HbA1c than fasting glucose [23]. However the assessment of glycemic control in diabetes by evaluating postprandial glucose levels has been seldomly used recently. The finding that postprandial glucose had a closer association with HbA1c [23] implies that postprandial glucose contributed to long-term glycemic control as, reflected in HbA1c as a measure of mean glycemic values in the previous 3 months.

The timing of measuring postprandial plasma glucose after the meal was controversial. To determine the impaired glucose tolerance for an oral glucose tolerance test, the adult patient was given a 75 g oral dose of glucose, and the plasma glucose level was then monitored. The American Diabetes Association implied that the peak value of postprandial glucose was 2 hours after the starting of the meal [24]. A previous study reported the close association of plasma glucose levels 2 hours after the administration of 75 g OGTT and 2 hours after a standardized meal [25]. Therefore, we measured the postprandial glucose level in the participants in our study 2 hours after a standard meal.

In some studies, postprandial glucose demonstrated a close relationship with cardiovascular disease or all-cause mortality, with a predicting ability that was better than the fasting glucose or HbA1c levels [26,27]. The possible pathophysiology relationship of postprandial hyperglycemia with cardiovascular disease has recognized that endothelial dysfunction plays an important role in the early phase of disease through the effects of oxidative stress [28,29]. Insulin resistance seemed to be an independent risk factor related to the inflammatory reaction associated with cardiovascular disease [30]. Insulin resistance leads to atherosclerosis mainly in the postprandial period rather than the fasting state, which only presents transient insulin resistance [31]. A recent study reported hyperglycemia-induced overproduction of superoxide released from the mitochondrial electron-transport chain was interpreted as arising from endothelial dysfunction [32].

An article published in the *Journal of Ophthalmology* in 2015 discussed the relationship between IOP and fasting glucose and postprandial glucose in the same populations [20]. However, in this study, all participants received blood testing to obtain the fasting glucose and postprandial glucose data, and subsequently, the intraocular pressure was measured. This is different from our study in that we measured IOP once, during the health check-up. In addition, the sample size of the study was relatively small. Multiple linear regression analysis in our report found a positive association between intraocular pressure and postprandial glucose, even after we adjusted for compounding factors. Despite the lack of a clear mechanism by which hyperglycemia causes elevated IOP, insulin resistance might provide an explanation for previous studies [21,33]. Other studies revealed that systemic arterial stiffness may account for the pathogenesis of increasing IOP in patients with diabetes mellitus with related macrovascular or microvascular complications [11]. However, other studies have found that genetic factors played a role in the elevated IOP in diabetes mellitus patients [9]. Furthermore, the autonomic dysfunction in diabetes has also been regarded as a contributing factor in increased IOP [34]. No matter the actual reasons for the IOP in diabetic patients, appropriate glycemic control and the maintenance of proper glycemic values should be emphasized, with our study highlighting the value of postprandial plasma glucose.

There are several limitations to our study. First, ours was a cross-sectional study; thus, we could not analyze the interaction between IOP and postprandial glucose due to the data being collected only once in a health check-up program, instead of with long-term follow up for observations. Second, IOP was measured only once for each eye unless the measurement of IOP was regarded as unreliable, in which case, we re-measured the IOP of both eyes. In view of the examining time and discomfort during the measurement of IOP, we did not measure the IOP of all the participants on many occasions and calculated the mean value. Third, the study population in our research was relatively robust, and the proportion of diabetes mellitus in the total participants was less than ten percent, although many participants were prediabetic. Furthermore, the general age in our study was relatively young, while aging is one of the important predisposing factors that affects the IOP [5]. Despite the fact we included age in our covariates for analysis, the significant association remained. The effect of elevated IOP related with aging was not present in our study owing to the relatively young population. In addition, the IOP measurement in our study use the non-contact tonometer (NCT) as the measuring tool rather than the Goldmann applanation tonometry (GAT) which has been considered the gold standard for measuring IOP. The main advantages of NCT over GAT are that they are more convenient, non-invasive and thus enhance patient cooperation. Another limitation was measuring postprandial glucose level with peripheral capillary blood instead of venous systems. However, the correlation coefficient between capillary and venous glucose levels was 0.911 [35]. Lastly, the value of central corneal thickness which was reported as an effecting factor for IOP was not measured [10]. A previous study demonstrated that IOP measurements should be adjusted for central corneal thickness, particularly in eyes with thin corneas [36]. Many eyes diagnosed with normal-tension glaucoma have thin corneas, which would be likely to lower the tonometrically measured IOP. Had we measured the central corneal thickness and made it a covariant in the multivariate analysis, the results would be much more credible.

## Conclusion

In conclusion, our study highlighted the finding that individuals with a higher level of postprandial glucose had a positive correlation with elevated intraocular pressure. Fluctuations in postprandial glucose may affect the aqueous humor production of the ciliary body or the aqueous humor outflow of trabecular meshwork. This may lead to systemic inflammation, and insulin resistance may promote microenvironmental perturbations that contribute to a predisposing milieu for elevated intraocular pressure or glaucoma. However, the association between postprandial glucose level and glaucoma warrants further longitudinal studies or trials to verify these findings for their application to clinical practice.

## Supporting Information

S1 File The minimal data set underlying the findings in our study. (SAV)
